# Do investors value the FDA orphan drug designation?

**DOI:** 10.1186/s13023-017-0665-6

**Published:** 2017-06-19

**Authors:** Kathleen L. Miller

**Affiliations:** 1 Office of Planning, Office of the Commissioner, US Food and Drug Administration, Silver Spring, MD USA; 2 Department of Health Policy and Management, Gillings School of Global Public Health, University of North Carolina at Chapel Hill, Chapel Hill, NC USA

**Keywords:** Orphan designation, Event study, Investors, FDA

## Abstract

**Background:**

The Orphan Drug Act is an important piece of legislation that uses financial incentives to encourage the development of drugs that treat rare diseases. This analysis studies the effects of a portion of the Orphan Drug Act, the orphan drug designation. Specifically, it studies the value that investors place on the orphan drug designation, by investigating how investors react to companies’ announcing that their product has received the designation.

**Results:**

The results, on average, show that the stock price of a company increases by 3.36% after the announcement of the designation, increasing the value of the company. The results are more pronounced for oncology drugs, and drugs being developed by the smallest companies.

**Conclusion:**

The orphan designation appears to be successful at generating positive value for companies, as seen by the positive and significant average increases in stock price.

**Electronic supplementary material:**

The online version of this article (doi:10.1186/s13023-017-0665-6) contains supplementary material, which is available to authorized users.

## Introduction

The orphan drug designation was created in 1983 when the Orphan Drug Act (ODA) was enacted by Congress, and is administered by the Office of Orphan Products Development at the Food and Drug Administration (FDA). The purpose of the designation was to create financial incentives for companies to develop new drugs and biologics for rare diseases. These incentives include a partial tax credit for clinical trial expenditures, waived user fees, and eligibility for 7 years of marketing exclusivity [1]. It was hoped that these financial incentives would lead to an increased number of orphan drugs coming to market. Since the legislation was passed, many studies of the effects of the ODA have been undertaken, including analyses of trends in orphan designation rates, new orphan drug approvals over time, and the assessment of the benefits that have accrued to patients with rare disease by the development of these drugs [2–10].

This study seeks to measure an alternative area of the ODA: how investors respond to the announcement of an orphan drug designation. The goal of this analysis is to obtain a proxy estimate for the value of the financial incentives of the Act. If investors (i.e., individuals or institutions who purchase shares of the pharmaceutical company’s stock) view the designation and associated financial incentives as a positive benefit that increases a company’s value, then there should be a corresponding increase in the company’s stock price after the announcement of a designation. The aim of this study is to determine whether there is a statistically significant change in a company’s stock price after the announcement of an orphan designation, and how the change may differ based on drug or company characteristics.

## Background

Event studies are a common analytical method in the financial and economics literature to analyze the impact of events (such as company-level announcements, market shocks, legislation, etc) on stock prices [11–14]. While it is a lesser known method for public policy analysis, event studies have been used to analyze other types of announcements by pharmaceutical companies, such as drug approvals [15–25].

Various event studies have been conducted to analyze how announcements by pharmaceutical companies influence stock price. For example, the approval of a new drug has been shown to boost stock prices by between 0.35 and 1.53%, and positive Phase III clinical trial results increase stock prices by 1.56% [17–20, 26]. The small effect for the most important step, approval itself, is best explained as indicating that events later in a drug’s life-cycle may cause smaller effects, because so much of the information on the likelihood of a drug’s approval was incorporated into the stock price at an earlier time [18].

One mechanism by which this information might be incorporated early is through the announcement of news events that provide a “signal” to investors that the drug may produce financial value in the future. For example, the fast-track designation is similar to the orphan drug designation, in that it is given to new indications of drugs (both approved and unapproved), must be applied for to FDA by the company, and must meet congressionally mandated criteria [27]. In order to receive the fast-track designation, the drug must treat a serious condition, and meet an unmet medical need [27]. However, unlike the orphan designation, the fast-track designation does not provide the company with any explicit financial benefits for developing its drug. (The benefits of a fast-track designation include the availability of more frequent meetings with the FDA, as well as the use of a rolling review when submitting a marketing application for a new drug.)

For the fast-track designation, the most recent event study estimated an average stock price increase of 6.25% after the announcement of the designation occurred [15]. Studies conducted in the earliest years of the program found increases of between 9 and 10% [16, 28]. These are both large effects, and seem to indicate that investors place strong, positive value on the information conveyed by the announcement of a fast-track designation, and therefore believe that it warrants an increase in the company’s value. Given that the orphan designation provides information, as well as tangible financial incentives, investors should react to its announcement in a similar manner to that of a fast-track designation. However, no previous studies of the orphan designation could be found.

## Study data and methods

### Analysis

Event studies typically define an “event” as a public announcement that affects either a single company or multiple companies simultaneously. For this analysis, the event is a company’s public announcement that it has received an orphan designation from the FDA for one of its drugs. [A full outline of the methods used for this study is available in the technical appendix found in the Additional file 1.] To estimate the size of the investor reaction to the announcement, this study uses the market model. First, a predicted value is created, which estimates the stock return that would be expected in the absence of the event. This value is then compared to the actual returns.

A predicted value of the company’s stock price on the day of the announcement is constructed using a set of ordinary least squares (OLS) regression models. The prediction utilizes the company’s daily stock prices in the 6 months leading up to the announcement, as well as the market returns from the S&P 500 composite index during the same period, to control for market-level fluctuations.

Once this prediction has been generated using the regression, it is compared to the actual stock price on the day of the announcement. The difference between these two numbers is the “abnormal return” of the stock, which is the value that investors place on the event—in this case, the announcement of the orphan designation. The abnormal returns are created for each event, and then summed to create the “cumulative abnormal returns” or CARs—which is the average value of the announcement of the orphan designation. The statistical significance of the CARs is determined using a nonparametric generalized rank t-test; specifically, the GRANK-T test [29].

The central analysis of this paper determines the magnitude and statistical significance of the CARs for the orphan designation announcement over the whole study period, 1983—2015. Additionally, to further investigate whether there are any differential effects based on drug type or company size, the data are sub-divided for two additional analyses. First, the observations are split by whether the drug is indicated for an oncology or non-oncology indication. A drug is determined to be for an oncology indication if the primary indication of the drug, for the orphan designation, is an oncology therapeutic. Palliative, supportive and diagnostic agents for cancer are not included in this category. All other indications are determined to be “non-oncology”. This analysis is also split longitudinally (periods 1985—2005 versus 2006—2015) to identify any trends over time, given the potentially changing nature of the drug development process.

Second, the observations are sub-divided by company size. Companies are classified into four categories using market capitalization (i.e., the current price of a share of a company’s stock multiplied by the number of outstanding shares) as a proxy for company size: less than $50 million, known as nano-cap companies; between $50 and $250 million known as micro-caps; between $250 million and $2 billion, known as small-caps; and, greater than $2 billion, known as mid-caps or large-caps.

### Data

The FDA orphan designation announcements were found via a systematic search of LexisNexis (a comprehensive news and legal database). A total of 1085 announcements were found, spanning the years 1985—2015. Four exclusion criteria were applied to the data, primarily to ensure that stock data existed for the observation, and to ensure that no confounding company-level events occurred simultaneously with the announcement. [A detailed description of the exclusion criteria can be found in the technical appendix in the Additional file 1.] The exclusion criteria removed a total of 762 observations, and the final sample size was 323 observations for the overall and oncology analyses. There was missing market-cap data for 76 companies, leaving a final sample size of 247 observations for the firm size analysis.

Stock data were obtained from the Center for Research in Securities Prices (CRSP), including both the data for individual companies, and for the S&P 500 Composite Index. Market capitalization data were obtained from Compustat.

### Limitations

The main limitation of this study is its limited generalizability to all companies that develop orphan drugs, because the study is only able to include products developed by companies that are listed on a US stock exchange. The study is therefore not necessarily generalizable to private companies, or companies traded outside of the US. A second limitation of this study is the small sample size of some of the analyses. While not a problem for the event study analysis, the sample sizes may limit the GRANK-T test’s ability to detect statistical significance.

## Results

The results of the event study are presented in Table 1. The CARs over the entire study period are 3.36%. This means that, on average, a company’s stock price increases by 3.36% after the announcement of an orphan designation over what it would have been had the announcement not occurred. The GRANK-T test statistic on this estimate is statistically significant at the 5% level.

**Table 1:** Results of Analyses

Study	Cumulative Abnormal Returns (CARs)	GRANK-T Test Statistic (*p*-value)
*Analysis 1* (*n* = 323)	3.36%	2.41 ^a^ (0.992)
*Analysis 2*		
Oncology (*n* = 169)	3.78%	2.07 ^a^ (0.976)
1985–2005 (*n* = 67)	3.47%	1.09 (0.862)
2006–2015 (*n* = 102)	3.98%	1.73 (0.958)
Non-oncology (*n* = 154)	2.91%	1.57 (0.942)
1985–2005 (*n* = 67)	3.28%	2.17 ^a^ (0.985)
2006–2015 (*n* = 87)	2.62%	0.86 (0.805)
*Analysis 3*		
Nano-cap: Market cap <$50 m (*n* = 40)	8.87%	1.02 (0.846)
Micro-cap: Market cap >$50 m & < $250 m (*n* = 94)	4.25%	1.50 (0.933)
Small-cap: Market cap >$250 m & < $2b (*n* = 92)	0.15%	0.40 (0.655)
Mid- & Large-cap: Market cap >$2b (*n* = 21)	−0.20%	−0.17 (0.433)

^a^statistically significant at the 5% level

In the second analysis, overall CARs for the oncology announcements are 3.78% (statistically significant at the 5% level) and are 2.91% (not statistically significant) for the non-oncology announcements. When these groups are split longitudinally, we find the CARs increase over time for the oncology orphan designations, and decrease for the non-oncology orphan designations. The CARs for the oncology announcements are 3.47% versus 3.98% for the earlier versus later period (neither is statistically significant). The CARs for the non-oncology announcements are 3.28% (statistically significant at the 5% level) versus 2.62% (not statistically significant) for the earlier versus later period.

Finally, in the third analysis, the CARs decrease significantly as the size of the market capitalization of the company increases. The CARs are 8.87% for the nano-caps, 4.25% for the micro-caps, 0.15% for the small-caps, and −0.20% for the mid- and large-caps. (None of these are statistically significant.)

Wilcoxon rank sum tests were used to examine whether the differences between any of the groups in the study were statistically significant. No statistical significance was found, although this may be due partially to the small sample sizes, as the magnitude of the differences are suggestive.

## Discussion

The success of the Orphan Drug Act (ODA) has been measured in numerous ways since it was enacted in 1983. The key purpose of the ODA is to use economic incentives to induce companies to develop drugs for rare diseases. This analysis seeks to understand whether investors in the pharmaceutical companies developing these drugs place a positive value on the orphan designation. To do this, the study uses positive stock price changes as a proxy measure for the value that investors place on the financial incentives of the Act. While the analysis cannot determine the exact value that investors place on the designation, nor the incentives that they value most, it can study the direction and magnitude of their attitudes toward the designation, and its corresponding financial incentives.

The study finds that investors in pharmaceutical companies view the orphan designation as a signal of higher company value. The overall CARs show an average increase in a company’s stock price of 3.36% after the announcement of an orphan designation (over what it was predicted to have been had the announcement not occurred). Negative CARs would have indicated that investors do not find economic value in the designation, and would potential dissuade companies from continuing to develop orphan drugs. However, the fact that large and positive CARs are found in the analysis implies that investors find value in the development of these drugs, which may lead to continued development.

The magnitude of the CARs in this study is almost double the maximum CARs that have been found in studies of drug approvals; however, it is almost half of what has been found in studies of the fast-track designation [8–14]. It is not surprising that the orphan designation produces higher CARs than a drug approval; orphan designation typically occurs early in the drug development process (sometimes even in the preclinical phase) and is therefore more valuable to investors because they have little other information regarding the drug and its likelihood of success.

However, it is surprising that the orphan designation produces only half the stock response of a fast-track designation. It may not be immediately intuitive why this should be true, given that both are FDA drug designations. The fast-track designation conveys no explicit financial benefits to companies that receive it, whereas the orphan drug designation does convey tangible financial benefits (both immediately and in the future). However, it is possible that investors’ reactions to the orphan designation are more tempered than their reaction to the fast-track designation because of the historically smaller potential for sales of most orphan drugs. Because orphan drugs, by definition, treat rare diseases, the number of potential patients who might purchase the drug is limited (although the high price of some orphan drugs in recent years is changing this paradigm) [30]. In contrast, drugs receiving the fast-track designation often have very large potential markets and, therefore, greater potential to be highly profitable for the firm and investors (i.e. increase the valuation multiple of these companies).

It is also possible that investor value is lower for orphan designations because it is conferred so early in the development process. It has been well documented that, on average, drugs in earlier stages of development are more likely to fail than drugs in later phases [31]. Therefore, it is possible that this lower valuation simply signals that orphan drugs, which tend to be in earlier stages of development when they are designated as compared with drugs receiving a fast-track designation, are viewed as more risky by investors (i.e. increase the discount rate of future earnings).

The results from the second analysis provide evidence that investors value oncology orphan drugs more than they do non-oncology orphan drugs. Over the entire study period, the oncology drugs have average CARs of 3.78% when the orphan designation was announced, as compared to 2.91% for non-oncology drugs. While the difference between these two results seems small in magnitude, a 0.8 percentage point (or approximately 30%) difference is sizable for an event study. It is not clear exactly what is driving this result. It is well known that oncology drugs are typically able to charge high prices and may also have non-orphan oncology markets that they can expand into, increasing their market size and therefore sales [32, 33]. However, this is also true for many non-oncology orphan drugs. It appears though, that when the data is broken out in this manner, investors believe that oncology orphan drugs will provide more value to companies (i.e. increase the projected earnings base).

The second analysis also shows a change in investor’s reactions to these orphan drugs over time, although it is less dramatic in magnitude than the overall comparison of oncology vs non-oncology drugs. Over the two time periods studied, 1985—2005 & 2006—2015, the CARs of the oncology group increase 15%, while the CARs of the non-oncology group decrease by 20%.

The results also show a striking difference in investor reactions to the orphan drug designation when broken out by company size. For the smallest companies, the nano-caps with less than $50 million in market cap, the CARs are almost 9%, while for the largest companies, with a market cap of greater than $10 billion, the CARs are negative 0.2%.

Overall, it is not surprising that smaller companies show higher gains than larger ones. Large companies may have multiple drugs under development, or have already approved drugs, which leads to more frequent announcements to investors, and a lower likelihood that any single announcement would lead to a significant change in stock price. However, nano- and micro-cap companies likely only have one product under development, and no approved drugs, so any information (positive or negative) regarding the product will be important information for investors (especially when projecting discounted future earnings of the companies).

### Policy implications

From a policy perspective, two main questions need to be answered in order to assess the success of the ODA. First, did the act actually result in new orphan drugs being developed? And second, is the overall cost of the program worth the benefits, in terms of output? This analysis explores part of the former question, by assessing whether investors in companies developing orphan drugs perceive these types of drugs as a good use of firm research dollars. The positive stock reaction to the announcement of orphan designations provides solid evidence that investors view research and development of orphan drugs to be of positive value to the company. Had negative returns been found, it would have signaled that investors believed that the companies would lose money developing these drugs, which in turn may have discouraged future investment. The positive signals by investors that were found indicate that companies will continue development in this space.

However, the results also show that the orphan designation does not seem to have a homogeneous incentive effect. It is clear that, at least in terms of stock gains, the greatest incentives fall to oncology drugs, and drugs being developed by the smallest companies.

This result is not necessarily a negative feature of the orphan designation. For instance, one of the main financial incentives of the ODA, the tax credits for clinical trials, can be used only if the company has made a profit, and therefore has tax liabilities. (The tax credit can also be carried forward for 20 years, so companies can use the credit when they begin making profits, and it may be partly transferred if they merge or are sold to a company with a tax liability.) As discussed, the nano- and micro-cap companies rarely have a drug on the market, and therefore have no profits. So this stock price increase may be an important non-explicit financial incentive to these companies.

The increased stock price incentive for the development of an orphan oncology drug versus other types of orphan drugs is worth noting. Given the difference in CARs, it may be that non-oncology drugs are implicitly receiving a lower value of financial incentives for their development. The seriousness of this difference is not readily apparent. This study cannot determine whether fewer non-oncology, rare disease drugs are being developed, or even whether the magnitude of this difference is significant to companies or their investors; however, it warrants further study.

## Conclusion

The purpose of this study was to study whether the Orphan Drug Act has been successful in incentivizing drug development for rare diseases, by analyzing the investor valuation of the orphan designation. The results indicate that the designation has been successful in this area: investors place positive, statistically significant, value on the orphan drug designation. These results were especially pronounced for oncology drugs and the smallest companies.

## Additional file

Additional file 1: Technical Appendix. (DOCX 70 kb)
