# Supplementary material for: Do investors value the FDA orphan drug designation?
Source: Orphanet J Rare Dis. 2017 Jun 19;12:114. doi: 10.1186/s13023-017-0665-6 (PMC5477091; doi:10.1186/s13023-017-0665-6)
Supplement: Additional file 1: — Technical Appendix. (DOCX 70 kb) [file 13023_2017_665_MOESM1_ESM.docx]

**Technical Appendix**

To evaluate whether investors perceive the orphan designation as an effective signal (i.e., adding positive value to a firm), this analysis utilizes an event study methodology. An “event” is typically defined as a publicly visible occurrence that affects either a single firm or multiple firms. For example, other events that have been used with this methodology included a firm announcing that its drug has been approved for market use, withdrawn from market use, or that one of its drugs received a n FDA drug designation. For this study, the “event” is defined as a firm publicly announcing that it has received an orphan designation. The event study first predicts what a firm’s stock price would have been at the time of the event, in the absence of the event occurring, which known as the expected return.^[[1]](#footnote-1)^ Next, the predicted stock price is subtracted from the actual stock price at the time of the event to find the abnormal return. The abnormal return is deemed to be the effect of the event on investor’s perceptions of the firm.

The event timeline includes three periods: the estimation window, the washout period, and the event window. The estimation window is defined as a period of time prior to the event and is used to estimate the expected return. This analysis uses the 120 trading days prior to the public announcement by a firm of the award of an orphan designation, through the 11 trading days prior to the event, as the estimation window (*t*= -121,-11). The washout period is defined as the 10 days before the event occurs (*t*= -10,-1), and is used to provide a buffer between the estimation window and event window, to ensure that the expected returns are not biased by any near-event occurrences. The event window is the period of time during which the effects of the event are expected to be reflected in the firm’s stock price. For this analysis, the event window is defined as the day of the event (*t*= 0) plus the day after the event (*t*= 1). The day after the announcement of the orphan designation is included in the event window to capture any residual reactions from investors (1).

To estimate the expected return of a stock consistent with the literature, this analysis uses the market model, which is an Ordinary Least Squares (OLS) regression model, and is expressed in Equation 1 (2-11). Because the data in this analysis have multiple instances of orphan designations on the same, or close, days, which could cause cross-correlation in the residuals, the market model is the best fit among the common models in the event study literature (5). (The model sensitivity analysis, presented later in this appendix, explores the model specification further.) In this model, $R_{i,t}$ and $R_{m,t}$ are the period *t* returns on the stock of firm $i$ and the representative market portfolio. Following previous research, the S&P 500 Composite Index is used as the respective market portfolio (7, 12-15). Conceptually, if a perfect linear relationship is expected between the market return and an individual stock return, $\alpha_{i}$ (the intercept term) and $\beta_{i}$ (the slope term) should have perfectly predicted the individual stock return, $R_{i,t}$; $\epsilon_{i,t}$, the error term, should therefore be mean zero. Unexplained deviations from this relationship are picked up by the error term, which is the basis for calculating the abnormal returns.

$$R_{i,t}= \alpha_{i}+\beta_{i}R_{m,t}+\epsilon_{i,t} (1)$$

The abnormal returns, $\hat{\epsilon}_{i,t}$ , are calculated using Equation 2 (10, 16, 17). The abnormal returns can be interpreted as the investors’ reaction to the event for the individual firm.

$$\hat{\epsilon}_{i,t}=R_{i,t}-\hat{a}_{i}-\hat{b}_{i}R_{m,t} (2)$$

Finally, the abnormal returns are aggregated by individual firms across time (as needed) and then aggregated across both firms and time; this number is known as the cumulative abnormal returns (CARs). The CARs are interpreted as the investors’ reactions to the event overall; the average reaction to the event over all firms.

*Significance Testing*

The statistical significance of the cumulative abnormal returns is determined using a GRANK-T test as outlined in Kolari and Pynnonen (2011) (5). As it has been shown that stock returns are not normally distributed, a standard t-test, which relies on the normal distribution, cannot be used to test for significance. Therefore, the GRANK-T test is used as a non-parametric, generalized rank, t-test. This test uses the respective rankings of the abnormal returns by firm, which generates an underlying distribution from which it conducts the significance test. This test is used for the overall test of significance of the CARs of the sample, as well as each individual test of significance for the oncology and firm size analyses.

*Exclusion Criteria*

A total of 1,085 announcements are found from the search of Lexis-Nexis, spanning the time period 1985-2015. To construct the final study sample, the following exclusion criteria are applied (Table 1).

First, designations for drugs that have been previously approved are excluded because they are likely inherently different from announcements for drugs which have not been approved. Approved drugs are much less uncertain to investors, as their safety and efficacy profiles are public and well understood. Additionally, the drugs have already cleared the largest hurdle in the eyes of investors: approval by the FDA. A total of 93 announcements are excluded for this reason.

Next, remaining firms are excluded if stock data are not available for the entire estimation and event windows. There are two possible reasons that the stock data are not available. The first is that the firm is a private company, and therefore stock information does not exist. The second is that data for the full time period are not available from CRSP. (There are many possible reasons for this, including the firm going public sometime during the time period, or getting acquired during the time period.) A total of 525 announcements are excluded due to this criterion.

Lastly, the remaining firms are excluded if another significant, potentially confounding, event occurs during the event window (*t*= 0, 1). This exclusion is necessary to ensure that the abnormal stock return estimates are not biased by other announcements occurring at the same time; that they only contain the stock reaction due to the orphan announcement. Observations are excluded if one of the following is announced during the event window: (a) any drug news (e.g., clinical trial results, patent received, drug approved, grant received, other designation received); (b) financing news (e.g., 8-K filed, royalty payment received, common stock offering, credit downgrade, debt financing); (c) leadership news (e.g., new leadership, leadership leaving, leadership optioning stock); (d) firm news (e.g., merger or acquisition announced or finalized, partnership deal announced, licensing deal announced). A total of 143 announcements are excluded due to this criterion.

Additionally, one announcement is excluded from the analyses because it is an outlier. In event studies, outliers can strongly affect the conclusions of the study, causing bias because they are inherently different from the other events. This outlier has an abnormal return of 97%, which is over eight standard deviations above the mean. It is also over three standard deviations above the next largest abnormal return.

In the analysis by firm size, 76 companies are excluded due to missing market capitalization data.

Once the criteria are applied, a total of 323 announcements remain, spanning the time period 1985-2015. A graph outlining the final distribution of announcements by year is presented in Figure 1. For the oncology analysis, there are 169 oncology therapeutic agents identified, leaving the remaining 154 announcements classified as non-oncology. In the 1985—2005 period, there are 67 oncology products, and the same number for non-oncology. In the 2006—2015 period, there are 102 oncology products and 87 non-oncology products. For the company size analysis, there are 40 companies classified as nano-cap (market cap less than $50 million), 94 classified as micro-cap (market cap between $50 and $250 million), 92 classified as small-cap (market cap between $250 million and $2 billion), and 21 classified as mid-cap or large-cap (market cap greater than $2 billion).

**Table 1. Exclusions and Sample Sizes**

| **Analysis or Exclusion** | **Sample Size for Each Analysis (Removed)** |
| --- | --- |
| ***Initial Sample*** | 1,085 |
| Exclusion 1: Previously approved | (93) |
| Exclusion 2: No stock data available | (525) |
| Exclusion 3: Confounding events | (143) |
| Exclusion 4: Outlier in abnormal returns | (1) |
|  |  |
| ***Aim 1*** Time period: 1985-2015 | 323 |
|  |  |
| ***Aim 2*** |  |
| Oncology | 169 |
| 1985-2005 | 67 |
| 2006-2015 | 102 |
|  |  |
| Non-oncology | 154 |
| 1985-2005 | 67 |
| 2006-2015 | 87 |
|  |  |
| ***Aim 3*** |  |
| Exclusion: No market cap data available | (76) |
| Nano-cap: Market cap <$50m | 40 |
| Micro-cap: Market cap >$50m & <$250m | 94 |
| Small-cap: Market cap >$250m & <$2b | 92 |
| Mid- & Large-cap: Market cap >$2b (max $283b) | 21 |
|  |  |

**Figure 1. Distribution of Announcements by Year, Final Sample**

*Model Sensitivity Analysis*

To judge the sensitivity of the results to model choice, two additional models are run for the main analysis: the Fama-French 3-Factor model, and the constant means model. The constant means model does not use any variables other than the predicted value of the stock to calculate the expected returns. The Fama-French model uses the market return, plus two additional variables (returns of small market cap minus big market cap firms, or SMB, and high book-to-market minus low book-to-market firms, or HML) to calculate the expected returns. SMB and HML values were retrieved from French’s website on February 29, 2016 (18). As seen in the manuscript, the market model returns CARs of 3.36% (statistically significant at the 5% level) (Table 2). The constant means model returns CARs of 3.43% (not statistically significant), and the Fama-French model returns CARs of 5.65% (statistically significant at the 5% level).

While both of these alternative models do have larger returns than the market model, neither reaches higher statistical significance. Due to this result, and because the market model has additional statistical properties that make it a better choice, it was the model chosen to be presented in the text. However, the model sensitivity results indicate that the results of this study may underestimate the true value of the CARs.

Table 1. Model Sensitivity Analysis Results

| Model | Results from Main Analysis | Grank-T Test Statistic |
| --- | --- | --- |
| Constant Means | 3.43% | 1.44 |
| Market (used in manuscript) | 3.36% | 2.41* |
| Fama-French | 5.65% | -2.10* |
| *statistically significant at the 5% level | | |

*Notes*

The Office of Orphan Products Development at the FDA regularly publicly publishes orphan designations on their website within a designation database. See: <https://www.accessdata.fda.gov/scripts/opdlisting/oopd/>

References

1. Miller KL, Nardinelli C, Pink G, Reiter K. The Signaling Effects of the US Food and Drug Administration Fast‐Track Designation. Managerial and Decision Economics. 2016.

2. Im KS, Dow KE, Grover V. Research report: a reexamination of IT investment and the market value of the firm—an event study methodology. Information systems research. 2001;12(1):103-17.

3. Alefantis TG, Kulkarni MS, Vora PP. Wealth Effects of Food and Drug Administration" Fast Track" Designation. Journal of Pharmaceutical Finance, Economics, and Policy 2004;13(3):41.

4. Dedman E, Lin SW-J, Prakash AJ, Chang C-H. Voluntary disclosure and its impact on share prices: Evidence from the UK biotechnology sector. Journal of Accounting and Public Policy. 2008;27(3):195-216.

5. Kolari JW, Pynnonen S. Nonparametric rank tests for event studies. Journal of Empirical Finance. 2011;18(5):953-71.

6. Sarkar SK, de Jong PJ. Market response to FDA announcements. The Quarterly Review of Economics and Finance. 2006;46(4):586-97.

7. Sharma A, Lacey N. Linking product development outcomes to market valuation of the firm: The case of the US pharmaceutical industry. Journal of Product Innovation Management. 2004;21(5):297-308.

8. Sturm A, Dowling MJ, Röder K. FDA Drug Approvals: Time Is Money! The Journal of Entrepreneurial Finance. 2007;12(2):23-54.

9. Ahmed P, Gardella J, Nanda S. Wealth effect of drug withdrawals on firms and their competitors. Financial Management. 2002:21-41.

10. Bosch JC, Lee I. Wealth effects of Food and Drug Administration (FDA) decisions. Managerial and Decision Economics. 1994;15(6):589-99.

11. Janney JJ, Folta TB. Signaling through private equity placements and its impact on the valuation of biotechnology firms. Journal of Business Venturing. 2003;18(3):361-80.

12. Austin DH. An event-study approach to measuring innovative output: The case of biotechnology. The American economic review. 1993;83(2):253-8.

13. Chesney M, Reshetar G, Karaman M. The impact of terrorism on financial markets: An empirical study. Journal of Banking & Finance. 2011;35(2):253-67.

14. Sood A, Tellis GJ. Do innovations really pay off? Total stock market returns to innovation. Marketing Science. 2009;28(3):442-56.

15. Stefanec NP. The impact of firm strategies on stock market value in the biotechnology industry. Applied Financial Economics. 2011;21(5):343-52.

16. Brown SJ, Warner JB. Using daily stock returns: The case of event studies. Journal of financial economics. 1985;14(1):3-31.

17. Campbell JY, Lo AW-C, MacKinlay AC. The econometrics of financial markets: princeton University press; 1997.

18. French, K. Fama/French 3 Factors. Retrieved February 29, 2016, from: <http://mba.tuck.dartmouth.edu/pages/faculty/ken.french/data_library.html>.

1. For this study, the closing price of the stock was used as the measure of ’stock price’, rather than an alternative measure, such as the bid price. The closing price is a standard measure of stock price in event studies, and is also the number that is used in calculating the market capitalization (which is a measure of the value of a company). [↑](#footnote-ref-1)
